# cleanSURFACES^®^ intervention reduces microbial activity on surfaces in a senior care facility

**DOI:** 10.3389/fcimb.2022.1040047

**Published:** 2022-11-09

**Authors:** Justin R. Wright, Truc T. Ly, Colin J. Brislawn, Jeremy R. Chen See, Samantha L. C. Anderson, Jordan T. Pellegrino, Logan Peachey, Christine Y. Walls, Jessica A. Bess, Anne L. Bailey, Katie E. Braun, Alexander J. Shope, Regina Lamendella

**Affiliations:** ^1^ Contamination Source Identification, Huntingdon, PA, United States; ^2^ AIONX, Hershey, PA, United States; ^3^ Veterans Health Administration Innovation Ecosystem, Washington, DC, United States; ^4^ Veterans Affairs Pittsburgh Healthcare System, Pittsburgh, PA, United States

**Keywords:** continuous cleaning, HAI, metatranscriptomics, long term care facilities, disinfection

## Abstract

As one of the top public health challenges outlined by the Centers for Disease Control (CDC), estimates report that hospital acquired infections (HAIs) claim the lives of 99,000 Americans and cost healthcare providers over $28 billion each year. In addition to underlying conditions related to age, elderly patients in long-term care facilities are at an elevated risk of acquiring HAIs. A large percentage of HAIs is attributable to contaminated surfaces and medical devices. To that end, this study utilized a metatranscriptomic sequencing workflow (CSI-Dx™) to profile active microbial communities from surfaces in the HJ Heinz Community Living Center, a long-term care facility in the Veterans Affairs Pittsburgh Health Care System. Swabs were collected from high-touch surfaces (Keyboard, Ledge, Workstation on Wheels, Worksurfaces) before (Baseline) and after cleanSURFACES^®^ were installed at 4 timepoints (Day 1, Day 7, Day 14, and Day 30). Microbial richness was significantly reduced after cleanSURFACES^®^ intervention (Wilcoxon test with Holm correction, p=0.000179). Beta diversity results revealed distinct clustering between Baseline and Post-intervention samples (Adonis, p<0.001). Reduction in bacterial (*Staphylococcus aureus, Staphylococcus epidermidis*, Staphylococcus *hominis*) and fungal (*Malassezia restricta, Candida albicans*, *Candida glabrata*, and *Candida orthopsilosis*) expression of opportunistic pathogens was observed. Additionally, a subset of taxa (*Corynebacterium*, *Cutibacterium acnes*, and *Ralstonia pickettii*) was present in specific Post-intervention timepoints and surface types. This study revealed decreased microbial activity, highlighting the potential for the combinatorial application of cleanSURFACES^®^ and regular decontamination practices to reduce the prevalence of microbes causing HAIs.

## Introduction

Despite advances in disinfectant products, infection control techniques and the best efforts of the healthcare industry, patients in U.S. healthcare facilities continue to acquire infections while receiving care. Recent estimates by the Centers for Disease Control (CDC) report that healthcare-associated infections (HAIs) claim the lives of 99,000 Americans each year and cost U.S. healthcare providers more than $28 billion in direct medical expenses and billions more in indirect costs (Stone, 2009). Due to underlying reasons including chronic illness and reduced immunological activity, elderly populations are known to be at a higher risk of acquiring HAIs compared to other age groups. Additionally, long term care facility (LTCF) patients are especially at risk of acquiring HAIs since residents require a higher degree of care and live in a confined environment. For instance, one study identified LTCFs yielded a higher magnitude of transmission for MRSA compared to hospitals (Cheng et al., 2013). The foremost common HAIs reported in LTCFs include urinary and respiratory tract infections, skin infections, and gastrointestinal infections (Haenen et al., 2019). In recent years, findings from epidemiological studies suggest LTCF residences are at an elevated risk of developing adverse health outcomes associated with COVID-19, ultimately emphasizing the importance of infection control policies in LTCF settings (Fisman et al., 2020; Romero et al., 2020).

One known vulnerability for infection control is the perpetual contamination of high-touch surfaces. Surfaces are typically disinfected with chemical-based cleaners, which have limited efficacy beyond the moment of cleaning (Edwards et al., 2020). Thus, surfaces are quickly re-contaminated and serve as reservoirs of pathogens until they are cleaned again (Scott, 2009; Attaway et al., 2012). These pathogens are then transmitted throughout common areas as caregivers, patients and visitors are consistently shedding microbes (up to 37 million microbes/hour) and seeding surfaces with a diversity of microbial taxa including potential pathogens (Bliznakova et al., 2007; Qian et al., 2012). As a result, as much as 40% of HAIs are attributable to contamination of environmental surfaces within healthcare facilities (Weber and Rutala, 2013). Because recontamination of surfaces can occur within minutes to days after disinfection events, efforts are underway to develop products to reduce surface contamination even as those surfaces are being used (Kwan et al., 2018; Edwards et al., 2020). The present study focuses on a unique approach: evaluating the efficacy of antimicrobial self-cleaning mats for use on select, high-touch surfaces in a LTCF. AIONX^®^ cleanSURFACES^®^ mats incorporate antimicrobial properties of silver and copper ions through a micro-electric current. Circuits are completed/closed when objects like microorganisms (i.e., pathogens and innocuous organisms) and droplets contact the mat. Subsequently, low concentrations of metal ions are released and create an environment that is toxic to microorganisms but harmless to human cells.

Several methods ranging from culture-based assays to next-generation sequencing (NGS) technology have been used to characterize microbes on surfaces (Sexton et al., 2011; Perry-Dow et al., 2022). Culture-based methods have been shown to be limited in their ability to identify accurately multiple microbes present in a sample (Gupta et al., 2019). However, other NGS technologies like shotgun metagenomics (MG) and metatranscriptomics (MT) are non-targeted approaches that reveal a more accurate representation of microbial community structure including non-culturable and even rare members of these complex microbial ecosystems (Shi et al., 2017). While MG is a comprehensive method that randomly sequences DNA to detect all microbes present in a sample, it is unable to differentiate between latent and active microbial communities. In clinically relevant settings, MT has been shown to be a promising method in its ability to identify active pathogens and antimicrobial-resistance mechanisms (Goswami et al., 2021; Malone et al., 2021).

This study builds upon previous research which used a novel MT sequencing workflow (CSI-Dx™) to profile active microbial communities of high-touch hospital intensive care unit (ICU) surfaces before and after the installation of cleanSURFACES^®^ mats (Chen See et al., 2021). The previous study focused on using the product in the ICU, where patients are at heightened risk of infection; however, due to reduced immunological activity and other potential risk factors like chronic illness, the elderly are also a vulnerable population and at an increased risk for developing HAIs (Katz and Roghmann, 2016; Zhao et al., 2019). Consequently, we utilized the same technology to assess the effectiveness of cleanSURFACES^®^ technology in a single LTCF at the Veterans Affairs Pittsburgh Healthcare System. Additionally, this study sought to incorporate a more optimal deployment of the test product to enhance the impact on pathogens, while reducing the cost to healthcare facilities were they to adopt the product.

## Methods

### Site information and sample collection

The current study was conducted at the HJ Heinz Community Living Center (Pittsburgh, PA), a Long-Term Care Facility that is among the 18 Veterans Integrated Services Networks (VISNs). The Veterans Administration Healthcare System divides the U.S. into 18 VISNs which serve as regional systems to supply care to veterans. Located in an urban setting, the HJ Heinz Community Living Center is a federal/non-profit organization that contains a total of 268 beds. This facility and the Pittsburgh VA Medical Center are located at separate sites.

A total of 84 environmental swabs were collected throughout the duration of the study (30 days). Swabs from each surface type and timepoint were collected using the same methodology described in (Chen See et al., 2021). Fifteen samples for each timepoint were collected. Within each timepoint, swabs were collected from 4 different high-touch surfaces. Surface types included keyboards, ledges, Workstation on Wheels (WoW) staging area keyboards, and nurse workstations. Surface types were selected based on findings from previous studies (Chen See et al., 2021), in conjunction with staff feedback on frequently touched or visited areas. A total of 15 Baseline samples were collected from individual quadrants at four timepoints for each surface type six hours after routine morning disinfection (Hour 0, Hour 2, Hour 4, and Hour 8). After Baseline samples were collected, cleanSURFACES^®^ mats were installed directly on top of Ledge and Workstation surface types. Since cleanSURFACES^®^ were unable to be installed directly on top of Keyboard and WoW Keyboard surface types, mats were placed underneath these respective surface types. Mats were divided into quadrants, where individual quadrants were sampled 6 hours post routine cleaning following 1, 7, 14, and 30 days after the installation of cleanSURFACES^®^. Quadrants of surfaces were sampled to reduce sampling bias since swabs have been shown to remove varying degrees of bioburden from surfaces (Landers et al., 2010). Routine cleaning by environmental services were performed based on CDC protocols for floors, trash, and high-touch surfaces with diluted 3M Disinfectant Cleaner RCT concentrate (CDC, 2019). These disinfection practices are identical to those that are performed on a regular basis at this institution. Environmental services and staff were instructed not to alter their disinfection practices throughout the duration of the study.

### RNA extraction, concentration, and quantification

As described in Chen See et al. (2021) RNA from swabs were isolated by extracting 1 mL of DNA/RNA Shield with a Zymobiomics DNA/RNA Miniprep kit (Zymo Research, Irvine CA). Samples from a single timepoint were extracted independently from other timepoints. Each extraction included a single no template control (NTC) to account for any introduced contamination. RNA extracts were concentrated using the Zymobiomics RNA Clean & Concentrator-5 kit (Zymo Research, Irvine CA), following manufacturer’s instructions. DNA concentrations from extracts were quantified using Quant-iT 1X dsDNA High Sensitivity Assay (Thermo Fisher Scientific, Waltham MA) to confirm DNase treatment. RNA concentrations were measured using Quant-iT RNA Assay (Thermo Fisher Scientific, Waltham MA). Each of the DNA and RNA quantification assays were performed on a Tecan Infinite 200 PRO plate reader (Tecan US, Morrisville NC). Extracts that were shown to have detectable concentrations of RNA were normalized to 500 pg input for downstream cDNA synthesis. However, if RNA concentration for extracts were below detection (< 0.25 ng/mL), the maximum possible volume of extract (8µL) was spiked in for cDNA synthesis.

### Library preparation and sequencing

As an input to the NEBNext Single Cell/Low Input RNA Library Prep Kit (New England Biolabs, Ipswich MA), concentrated RNA for each sample was spiked with 10 pg of a synthetic RNA construct (ERCC) to serve as an internal control (Pine et al., 2016). Libraries were prepared following the manufacturer’s protocol. Samples were quantified after library preparation using a Quant-iT 1X dsDNA High Sensitivity Assay (Thermo Fisher Scientific, Waltham MA). Once samples were quantified, a sequencing library was prepared by combining an equivalent mass from all sample libraries. Sequencing libraries were purified using Ampure XP beads (0.9X ratio) (Beckman Coulter, Brea, CA) and subsequently quantified using a Qubit 1X dsDNA HS assay (Thermo Fisher Scientific, Waltham MA). Purified sequencing libraries were diluted, denatured, and sequenced on an Illumina NextSeq 550 using a 150 cycle High Output v2.5 kit according to the manufacturer’s protocol.

### Bioinformatics methods and analysis

Upon completion of all sequencing runs, raw data were filtered and annotated using CSI’s *Rapid Active Pathogen Identification and Detection (RAPID-Dx* ^®^) pipeline as described in Chen See et al. (2021). To reduce noise due to index hopping, singletons and doubletons were removed from the summarized taxonomy annotation table on a per-sample basis. Post noise filtration, normalization was used to scale taxa frequencies proportionally to total microbial reads observed in a sample (See **Table 1** samples included in analysis). To confirm consistent library preparation and sequencing success as previously published (Chen See et al., 2021) the total number of annotated microbial sequences per sample were compared between the two studies using a Wilcoxon test with a cutoff of 0.05 for significance (**Supplemental Table 1**.) Downstream alpha diversity, beta diversity, and biomarker analyses were conducted as previously detailed in Chen See et al. (2021), with the following exceptions: per-sample normalization was performed using proportional scaling to the total number of microbial reads, and the unweighted Jaccard diversity metric was utilized to calculate both distances between samples and also contributions due to nestedness and turnover. Additionally, Spearman’s rank correlation was run to determine the relationship between normalized microbial genera associated with HAIs and days post intervention, with Holm’s correction applied to control the familywise error rate.

**Table 1:** Number of samples (n) per surface type and timepoint included in bioinformatic analysis after quality filtration.

	Timepoint				
Surface type	Baseline	AIONX^®^, Day 1	AIONX^®^, Day 7	AIONX^®^, Day 14	AIONX^®^, Day 30
**Keyboard**	4	4	4	4	4
**Ledge**	3	3	3	3	3
**Work Surface**	3	3	3	3	3
**Wow**	5	5	5	5	4
**Total**	15	15	15	15	14

## Results

In total, 84,975,691 raw sequences were generated across all 74 samples used in downstream analysis, representing 5 timepoints (Baseline, Day 1, Day 7, Day 14, and Day 30) and four surfaces (Keyboard, Ledge, Work Surface, and WoW Keyboard). Notably, the number of annotated microbial sequences in this study did not differ significantly from CSI’s prior cleanSURFACES^®^ study (Wilcoxon Rank Sum, p=0.623), and the mean raw sequence count was greater than 1.4 million reads per sample for both studies, suggesting ample coverage of microbial diversity **(** **Supplemental Table 2**).

### Alpha and beta diversity

**Figure 1:**
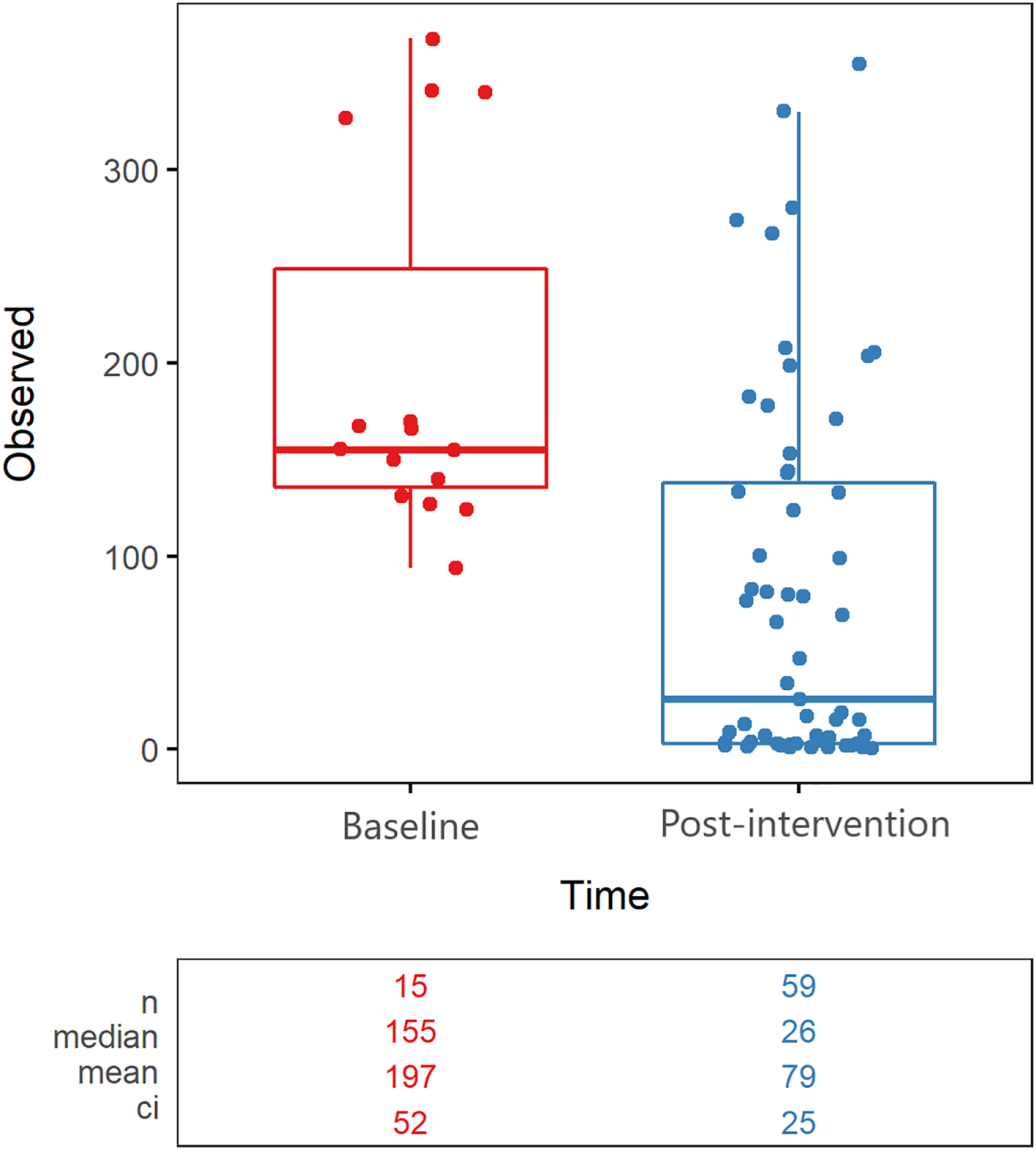
Alpha Diversity boxplot compares observed taxonomic richness between surface swabs collected prior to (red) and after (blue) the intervention of AIONX^®^ cleanSURFACES^®^. A significant decrease in observed species richness is observed after the intervention of the AIONX^®^ cleanSURFACES^®^ (p=0.000179). The number of unique taxa observed in each sample is reported, along with summary statistics for each group.

Overall microbial community richness was significantly lower (Wilcoxon test with Holm correction, p=0.000179) after cleanSURFACES^®^ application (mean richness = 279 observed species) in comparison to Baseline samples (mean richness = 126 observed species) (**Figure 1**). This difference was driven by samples collected on Day 14 and Day 30 (Wilcoxon test with Holm correction, p < 0.00001*), as Day 1 (Wilcoxon test with Holm correction, p = 0.79) and Day 7 (Wilcoxon test with Holm correction, p=0.058) richness did not differ significantly from Baseline (**Supplemental Figure 1**). Samples from Day 14 and Day 30 also had lower richness for each of the different surfaces (**Supplemental Figure 2**). Within the individual surface types, Keyboard and WoW Keyboard samples had significantly (Wilcoxon test with Holm correction, p=0.0274 and p=0.0274) lower richness after cleanSURFACES^®^ application. Active microbial community composition also differed significantly (Adonis, p<0.001) between Baseline and Post-cleanSURFACES^®^ samples (**Figure 1**). When comparing differences between samples during the intervention, 76.7% of the mean Jaccard distance between samples could be attributed to nestedness, meaning that loss of taxa was the primary factor differentiating samples in the study (**Supplemental Table 3**).

### LEfSe results

A total of 10 transcriptionally active microbial taxa (domains, phyla, genera, and species) were identified as being significantly elevated (Kruskal-Wallis p ≤ 0.05, log (LDA)≥1.5) in Baseline samples compared to Post-intervention samples (**Figure 5**). The *Staphylococcus*, *Streptococcus*, *Herbaspirillum*, and *Malassezia* genera were all significantly elevated within Baseline samples (Kruskal-Wallis p ≤ 0.05, log (LDA)≥1.5) Notably, there were significantly more transcripts representing the Bacteria and Eukarya domains in the Baseline and Post-intervention comparison (Kruskal-Wallisp ≤ 0.05, log (LDA) ≥1.5). Timepoint comparisons revealed that Day 1, Day 7 and Day 30 samples had several differential taxa (**Supplemental Figure 3**). In particular, *Acinetobacter*, *Ralstonia*, *Pseudomonas*, and *Cutibacterium* were among the differential taxa observed from Post-intervention timepoints (Day 1, Day 7, and Day 30) (Kruskal-Wallis p ≤ 0.05, log (LDA)≥1.5). All timepoint comparisons identified more taxa with increased activity in Baseline compared to Post-intervention samples, suggesting cleanSURFACES^®^ application reduced the global expression of microbial taxa on surfaces tested.

### HAI taxa correlations and boxplots

This study revealed a significant reduction in the prevalence of several genera associated with HAIs over the course of time (**Table 2**). Specifically, members of the *Candida* (r = -0.63, adj p <0.0001^*^)*, Staphylococcus (*r = -0.57, adj p = 1.40e^-6^), Enterococcus *(*r = -0.49, adj p = 5.16e^-5^), *Pseudomonas (*r = -0.33, adj p = 9.28e^-3^), and *Acinetobacter* (r = -0.39, adj p = 2.67e^-3^) genera all yielded significant negative correlations with time. HAI associated genera boxplots over time revealed clear reductions in taxa abundance over the course of the study (**Figure 3**). Members of the *Enterococcus* spp. and *Candida* spp. genera were both undetected by Day 14 and remained to be undetected by the final Day 30 timepoint. Conversely, *Cutibacterium* spp. were found to yield increased normalized abundance counts in subsets of Day 14 and Day 30 samples when compared to Baseline, Day 1, and Day 7 samples. Species level normalized abundance boxplots of annotated coagulase-negative *Staphylococcus* (CONS)*, Pseudomonas aeruginosa, Acinetobacter baumannii, Candida glabrata, Candida albicans, and Candida orthopsilosis* revealed a consistent trend of reduced pathogenic taxon abundance by Day 30 of the investigation (**Figure 4**; **Supplemental Figure 5**). CSI’s RAPID-Dx^®^ bioinformatic pipeline searches for active transcripts associated with viruses, and there were not any sequences within this dataset that were found to be annotated as a virus. Specifically, there were no reads annotated as SARS-CoV-2, Norovirus, Influenza, Enterovirus, Cytomegalovirus, Coxsackievirus, *Astroviridae*, or *Parvovirinae*.

**Table 2:** Genera associated with Hospital acquired infections (HAIs) Spearmans’s correlation results when comparing normalized abundance against days post Baseline.

Genus	Spearman’s rho	p	p.adj
**Candida**	-0.63	0.00e+00	0.00e+00
**Staphylococcus**	-0.57	3.00e-07	1.40e-06
**Enterococcus**	-0.49	1.29e-05	5.16e-05
**Acinetobacter**	-0.39	8.90e-04	2.67e-03
**Pseudomonas**	-0.33	4.64e-03	9.28e-03

## Discussion

Infections remain one of the leading causes of morbidity and mortality for patients in LTCFs. The most common types of HAIs reported from LTCFs include urinary, respiratory tract, skin, and gastrointestinal infections. More recently, a comprehensive European LTCF survey revealed LTCFs to have an estimated annual burden for SSTIs (skin and soft tissue infections) to be five times greater than acute care facilities (Suetens et al., 2018; Wilcox and Dryden, 2021). These studies ultimately highlight the importance of effective infection prevention policies and procedures, especially in a LTCF. To that end, this study assessed the effectiveness of cleanSURFACES^®^ mats applied within the HJ Heinz Community Living Center of the Veterans Administration Healthcare System. Our previous study evaluated the efficacy of the same technology in an ICU at UPMC Harrisburg (Chen See et al., 2021), and consequently, we sought to determine whether the same trends were observed.

### Alpha and beta diversity distinction between timepoints

**Figure 2:**
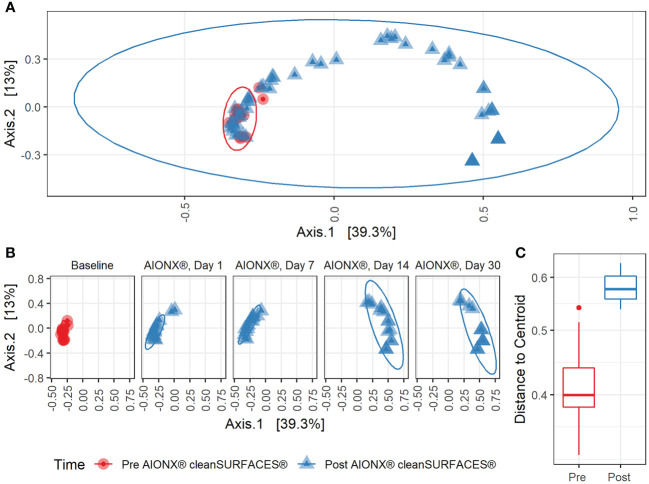
Beta Diversity during cleanSURFACES^®^ Intervention. Principal coordinates analysis (PCoA) of binary Jaccard distances between samples **(A)** before (red) and after (blue) the intervention of the AIONX^®^ cleanSURFACES^®^. **(B)** Matched principal coordinates analysis stratified by day reveals significant shifts in microbial community composition between samples when grouped by sampling day (adonis p < 0.001). **(C)** Boxplot comparing within-group unweighted jaccard distance dispersion before (red) and after (blue) the intervention of the AIONX^®^ cleanSURFACES^®^.

Alpha diversity results revealed that richness for Post-intervention timepoints (Day 7, Day 14, and Day 30) were significantly reduced compared to Baseline samples (**Figure 1**). The same trend was also observed for several surface types including, Keyboards, WoW Keyboards, and Work-surfaces (**Supplemental Figure 2**). This trend of reduced observed richness between timepoints and surface types had also been observed in our previous study (Chen See et al., 2021). However, the findings from the current study revealed a significant decrease in microbial richness Post-intervention (Day 14 and Day 30). This significant reduction in richness indicates that samples from the last two timepoints did not contain as many microbial taxa compared to other timepoints. In addition to differences in observed richness, microbial community composition between Baseline and Post-intervention samples were shown to be distinct from one another (Adonis, p<0.001) (**Figure 2**). As observed in the previous study, post-intervention timepoint (Day 1, Day 7, Day 14, and Day 30) samples are similarly shown to be widely dispersed compared to Baseline samples, which are compact to a single area (Chen See et al., 2021). This suggests that the microbial communities from post-intervention timepoints, especially for Day 14 and Day 30, were compositionally distinct from Baseline samples. In support of our findings, distinct microbial communities from healthcare surfaces before and after disinfection have also been observed (Perry-Dow et al., 2022).

### Reduction of opportunistic pathogens

**Figure 3:**
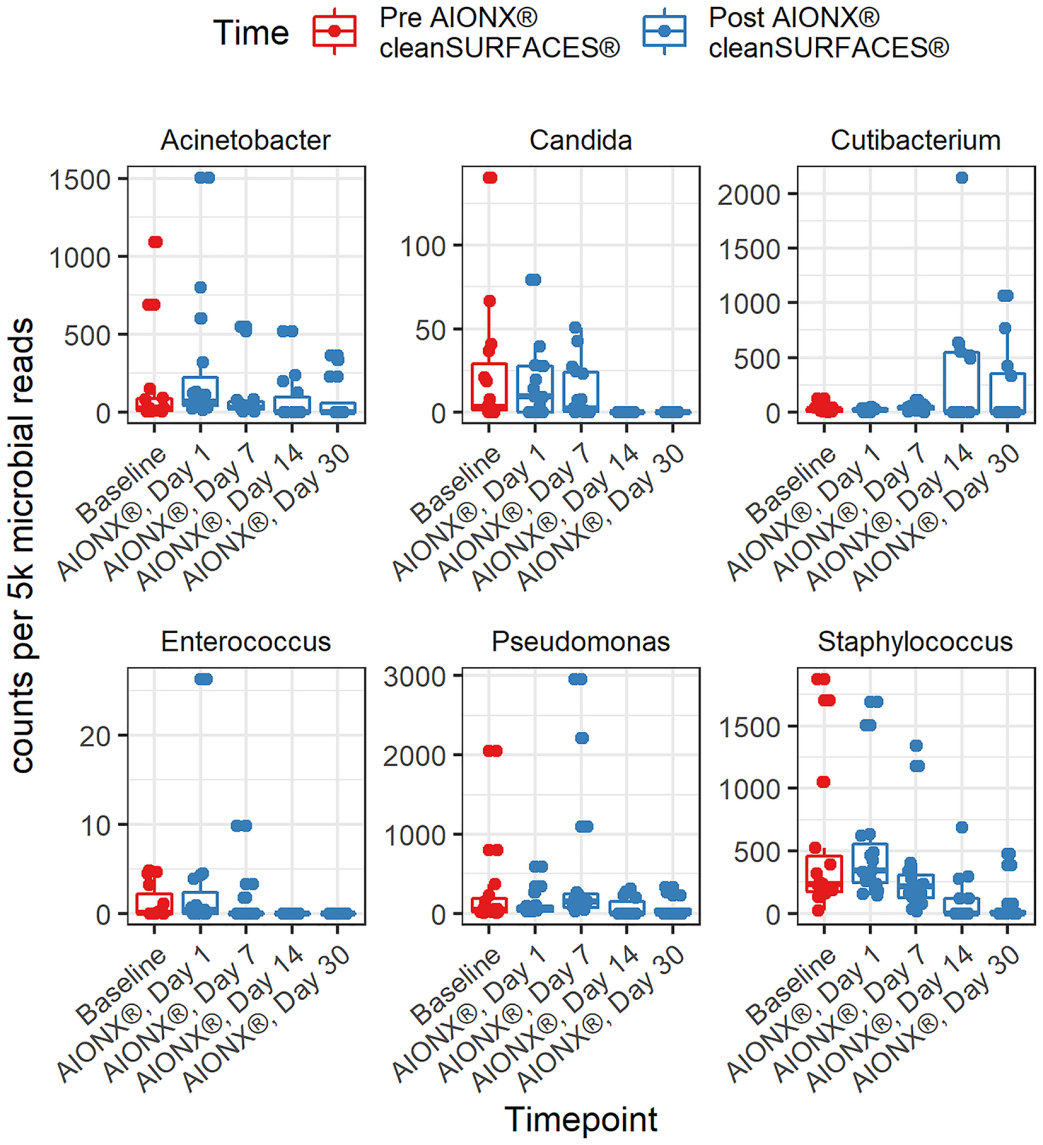
Normalized counts of genera associated with Hospital Acquired Infections, during cleanSURFACES^®^ intervention.

**Figure 4:**
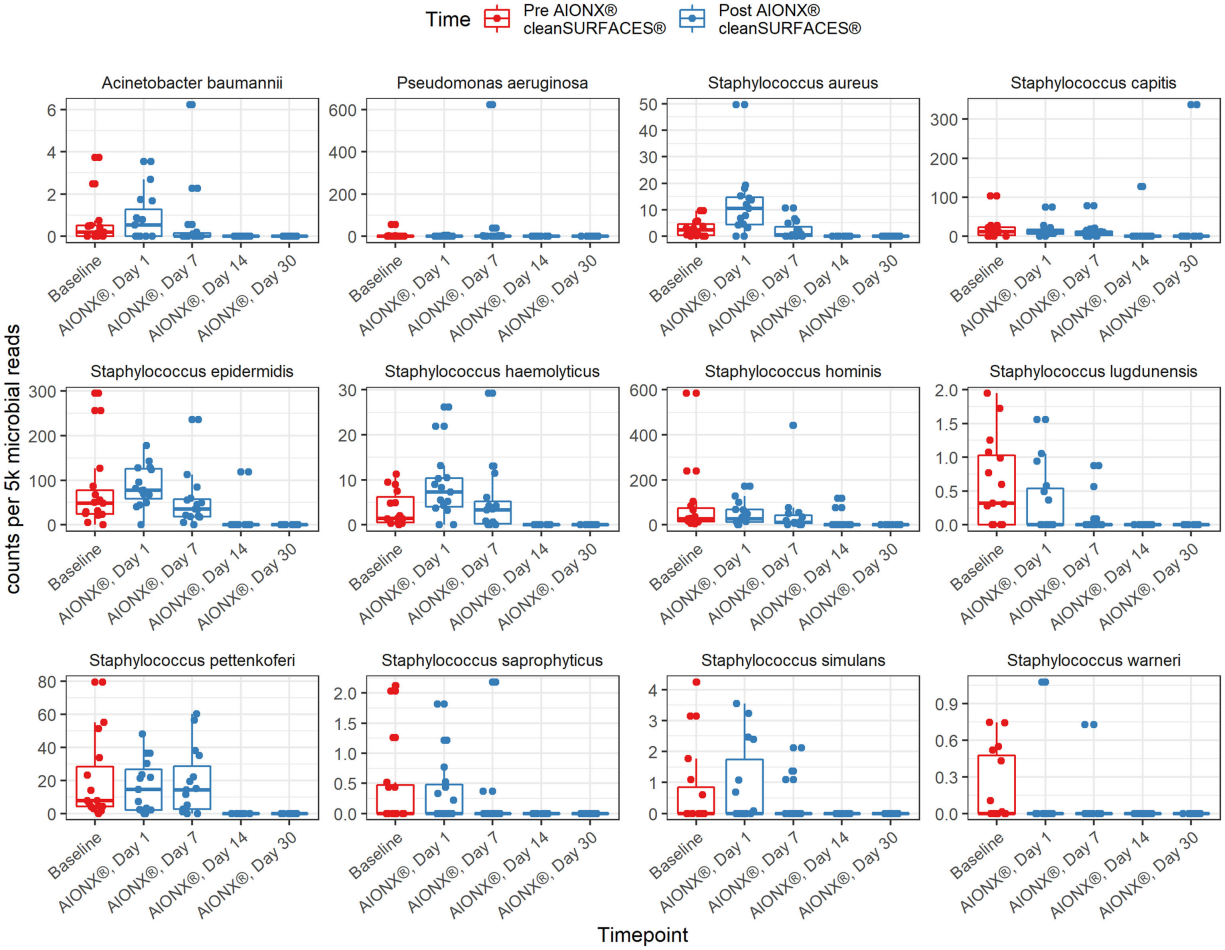
Normalized counts of species associated with Hospital Acquired Infections, during cleanSURFACES^®^ intervention.

At the genus and species levels, multiple pathogens of interest exhibited fewer annotated transcripts after the application of cleanSURFACES^®^ (**Figures 3**, **4**; **Supplemental Figures 3–5**). *Staphylococcus* was found to have less annotated transcripts among post-intervention timepoints compared to Baseline (**Figure 3**). Further resolution down to the species level revealed several *Staphylococcus* spp. to be reduced among post-intervention timepoint samples (**Figure 4**). This included a number of opportunistic pathogens commonly associated with HAIs, *Staphylococcus aureus, Staphylococcus epidermidis*, Staphylococcus *hominis*, and *Staphylococcus haemolyticus* (Otto, 2009; Jiang et al., 2012; Sielaff et al., 2016; Pérez-Montarelo et al., 2017). In concordance to these findings, reduction of several *Staphylococcus* spp. between Baseline and Post-intervention samples was also observed in the previous study (Chen See et al., 2021). Additionally, *Acinetobacter* spp. were also found to be present among Baseline and Day 1 samples (**Figure 4**; **Supplemental Figure 3**). Although *Acinetobacter baumannii* are ubiquitous in the environment, they are a known opportunistic pathogen and have been associated with HAIs among immunocompromised patients (Fournier et al., 2006; Al Atrouni et al., 2016; Reyes et al., 2020). The types of HAIs *Acinetobacter* spp. have been associated with range from urinary and respiratory tract infections to bacteremia (Peleg et al., 2008; Tavares et al., 2020). The reduction of these important opportunistic pathogens in both studies suggests that these mats, in conjunction with regular disinfection practices, could help prevent HAIs associated with various opportunistic pathogens over time.

**Figure 5:**
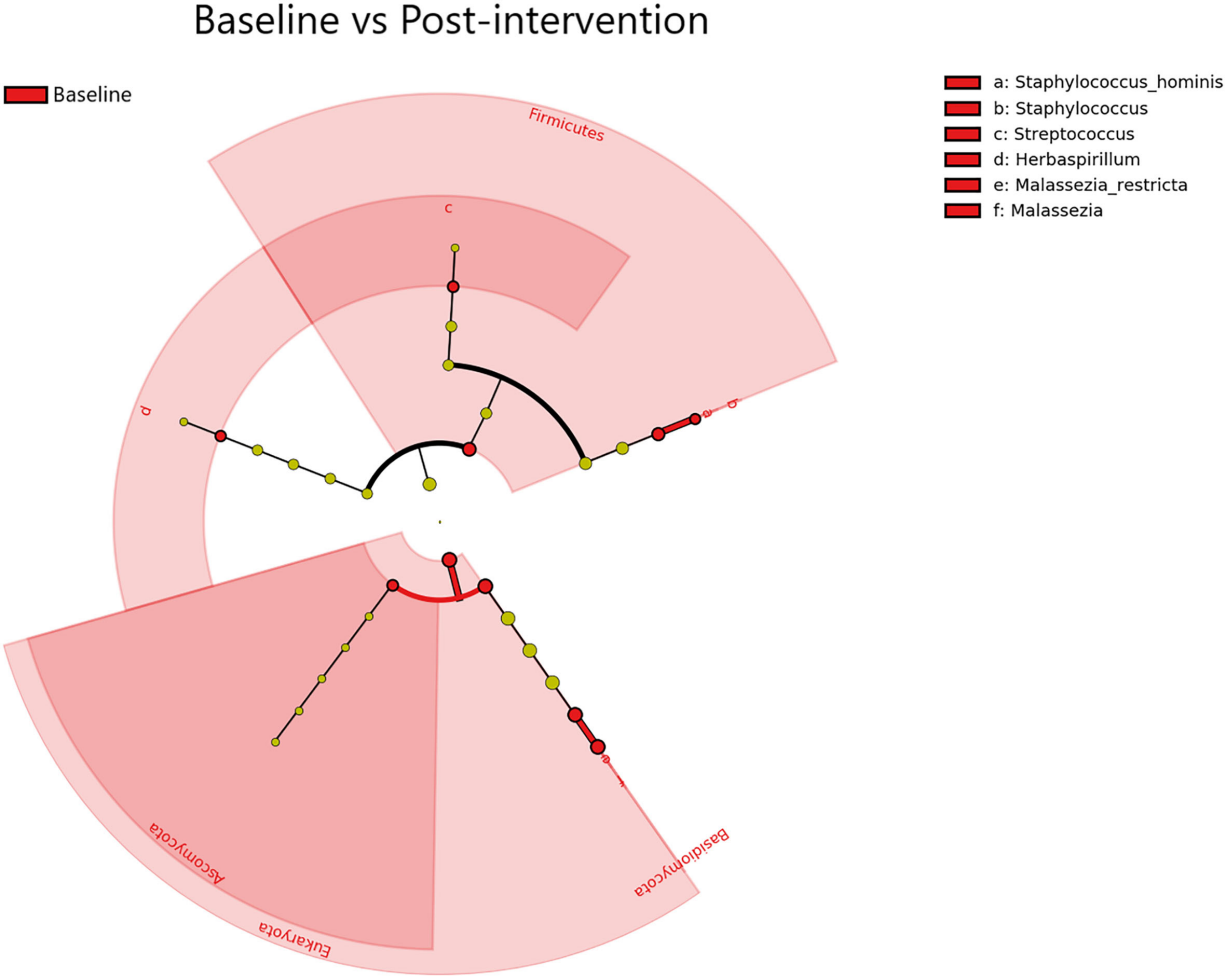
Differential Taxa (Domain, Phylum, Genus, and Species levels) identified by LEfSe Pre- vs Post-AIONX^®^ cleanSURFACES^®^ intervention analysis. The taxa shown were significantly (Kruskal-Wallis, p ≤ 0.05 and log (LDA)≥1.5) more active in Baseline samples. No enriched taxa were identified in the Post-AIONX^®^ cleanSURFACES^®^ group.

Annotated transcripts of several important fungal opportunistic pathogens were reduced on surfaces after the installation of cleanSURFACES^®^ mats. These fungal species included, *Malassezia restricta*, and several *Candida* spp. (*Candida albicans*, *Candida glabrata*, and *Candida orthopsilosis*). Overall, transcriptional activity of the skin commensal, *M. restricta*, was reduced after the cleanSURFACES^®^ intervention (**Figure 5**; **Supplemental Figures 3, 4**). Interestingly, *M. restricta* was found to be one of the three main drivers of sample clustering in the previous study (Chen See et al., 2021). As an opportunistic fungal pathogen, *M. restricta* has been associated with causing several skin conditions (Saunte et al., 2020) and more recently, infective endocarditis (Houhamdi-Hammou et al., 2021). Although all three *Candida* spp. have been found to cause bacteremia (Barbedo et al., 2015; Barchiesi et al., 2016), *C. albicans* is among the most common cause of hospital-acquired multi-drug resistant fungal infections (Kullberg and Arendrup, 2015; Arendrup and Patterson, 2017). Interestingly, while *C. glabrata* was previously thought to be a non-pathogenic fungal species, there has been an emergence of this species isolated from Candidiasis cases especially among immunocompromised patients (Fidel et al., 1999). Despite being closely related to *Candida parapsilosis*, a common cause of HAIs among immunocompromised patients and neonates, studies have found *C. orthopsilosis* to be less virulent (Tavanti et al., 2005; Feng et al., 2012; Barchiesi et al., 2016; Park et al., 2020). Though it is generally not resistant to fungicides (Brilhante et al., 2018; Arastehfar et al., 2019), one study found 40% of their isolates were resistant to azole fungicides (Rizzato et al., 2018). While most *Candida*-associated infections are believed to emerge from endogenous sources, recent studies have found that spread of fungal opportunistic pathogens also occur through the hands of healthcare workers (Shi et al., 2017; Arastehfar et al., 2019). Our results suggest that regular disinfecting protocols in conjunction with cleanSURFACES^®^ have potential in decreasing the activity of not only bacteria but fungi as well on high-touch surfaces in a hospital setting.

### Remaining microbes on surfaces after the installation of cleanSURFACES^®^

As observed in the previous study, several microbes were also identified to be more active post cleanSURFACES^®^ intervention. The following genus and species level microbes were identified on surfaces after the intervention, *Pseudomonas, Cutibacterium acnes*, and *Ralstonia pickettii* (**Figures 3**, **4**; **Supplemental Figure 3**). Although *Pseudomonas* was unable to be identified down to the species level, biomarker analysis revealed that *Pseudomonas* was enriched among Baseline samples. Subsequent comparisons by timepoints also yielded *Pseudomonas* spp. to be enriched in Day 7 samples as well (**Supplemental Figure 3**). Although members of the *Pseudomonas* spp. genus are diverse, Pseudomonas *aeruginosa* remains one of the most clinically-relevant *Pseudomonas* sp. (Driscoll et al., 2007). While this species has been found in low abundances in most human or animal impacted environments (Diggle and Whiteley, 2020), it is also regarded as an important cause of hospital-acquired infections (Blanc et al., 2007; Driscoll et al., 2007). In the previous study, *P. aeruginosa* was reduced following cleanSURFACES^®^ application but not enough to be statistically significant (Chen See et al., 2021). This suggests that there is an underlying microbial community of commensal microbes (i.e., *Pseudomonas* spp., *C. acnes*, and *R. pickettii*) that are likely being re-introduced by building inhabitants despite the presence of cleanSURFACES^®^ in conjunction with daily disinfection practices over time.

Two species level taxa (*C. acnes* and *R. pickettii*) were also present among post-intervention samples (**Figure 4**; **Supplemental Figure 3**). Additional biomarker analysis identified *C. acnes* to be significantly enriched among Day 30 samples. In concordance with these results, the genus, *Cutibacterium* spp., was also detected in Day 14 and Day 30 samples (**Figure 3**). Interestingly, this species was previously identified as being significantly less active after cleanSURFACES^®^ application (Chen See et al., 2021), though it was found to be enriched in Day 1 Ledge samples. Despite being a major skin commensal, *C. acnes* have also been found to be associated with causing shoulder prosthetic joint infections (PJI) and other skin conditions (Elston et al., 2019; Park et al., 2020). The presence of this opportunistic pathogen on surfaces towards the end of study could have been attributed to the fact that highly abundant skin microbes, like *C. acnes*, are continuously being shed in the environment (Hospodsky et al., 2012). Additionally, this finding could also suggest that the efficacy of cleanSURFACES^®^ begin to diminish within a month of installation. This potential limitation has also been previously discussed by Chen See et al. (2021). Biomarker analyses revealed *R. pickettii* to be significantly enriched among Day 7 surface samples (**Supplemental Figure 3**). While *R. pickettii* is generally isolated from moist environmental sources (i.e., lakes and rivers), they have been recently associated with nosocomial outbreaks of bacteremia (Kimura et al., 2005; Ryan and Adley, 2013). Interestingly, the sources of nosocomial outbreaks associated with *R. pickettii* have been attributed to contaminated medical solutions and disinfectants (Kimura et al., 2005; Weber et al., 2007). Although our results suggest that cleanSURFACES^®^ can reduce the activity of certain microbes, the microbes that remain active on high-touch surfaces have been found to be associated with various types of infections among immunocompromised patients, which includes the elderly (Nasir et al., 2019; Abdullah et al., 2021). Despite a subset of taxa was found to be enriched post cleanSURFACES^®^ intervention, an overall trend of decreased microbial activity was still observed in our study. This highlights the potential for cleanSURFACES^®^ in conjunction with regular decontamination practices to reduce the prevalence of microbes associated with causing HAIs. Though the prevalence of HAIs in the United States had decreased according to CDC’s most recent hospital survey (Magill et al., 2018), it is still estimated that one in thirty-one hospital patients suffer from HAIs (CDC, 2021a). Furthermore, HAIs continue to pose a large economic burden, directly costing hospitals in the United States an estimated $28.4 billion yearly (CDC, 2021b). Both the WHO and UK Royal College of Nursing emphasize the importance of good hand hygiene to prevent HAIs (Pittet et al., 2006; Sunley et al., 2017). Considering the results from the current study, cleanSURFACES^®^ mats could serve as an additional method to promote sanitation and prevent HAIs.

## Limitations and challenges

There were several limitations in this study. The most significant limitation was that this study was conducted in a single LTCF which may reduce the generalizability of our findings. While the study utilized metatranscriptomics to assess shifts in active taxa, shifts in functional genes were not investigated. Therefore, this study cannot comment on questions about functional genes (i.e., antimicrobial susceptibility patterns). Additionally, this study did not include matched controls at each timepoint. The presence of a functional cleanSURFACES^®^ mat would add bias into the entire network associated with the Long-Term Care Unit, ultimately posing a challenge in creating a true study control. Instead, we compared post-cleanSURFACES^®^ intervention samples to the samples taken before the mats were installed (Baseline). Due to the lack of matched controls, we were limited in our ability to definitively determine if the mats were the principal factor for all observed differences; however, hospital staff were instructed to not change their standard cleaning habits during the cleanSURFACES^®^ intervention. Although hospital staff were instructed to not change their behavior in how they interacted with high-touch surfaces during the investigation, variations in interaction could have occurred. For instance, the presence of cleanSURFACES^®^ mats could have impacted how often staff cleaned their hands and disinfected high-touch surfaces. Additionally, the sample size for more granular comparisons, like surface type per timepoint, was low, yielding between 3-5 samples for a given surface type at a timepoint.

## Conclusions

This study builds upon the previous study, as it assessed the same continuous cleaning technology in an additional healthcare facility. As before, we observed reduction in the overall number of active taxa after applying the cleanSURFACES^®^ mats in a single LTCF. Moreover, we noted decreased activity for several clinically important microbes, including *Pseudomonas aeruginosa* and *Candida parapsilosis*. Therefore, our findings provide additional evidence that this technology could complement existing sanitation protocols and technology to help prevent HAIs, especially in areas of high risk such as ICUs and LTCFs. Future studies in this area can utilize metatranscriptomic technology to assess shifts in active microbes and functional genes. In addition to functional gene analysis, future studies can also consider culturing and testing for antimicrobial susceptibility patterns to elucidate the mechanism by which certain microorganisms remain on disinfected surfaces. Additionally, future studies can also evaluate the clinical impact of cleanSURFACES^®^ on HAIs.

## Data availability statement

The datasets presented in this study can be found in online repositories. The names of the repository/repositories and accession number(s) can be found below: https://www.ncbi.nlm.nih.gov/, PRJNA799364. 

## Author contributions

RL, AS, and JW conceived and designed the experiments. KB and JB coordinated sample collection. JB collected samples. TL, CW, SA, JP, and LP processed the samples for sequencing. RL, JCS, CB, TL, and JW analyzed the data. All authors contributed to writing and revising the manuscript. All authors read and approved the final version of the manuscript.

## Funding

This material is based on research sponsored by Air Force Research Laboratory under Agreement Number FA8650-20-2-5506 in support of Air Force Research Laboratory. The U.S. Government is authorized to reproduce and distribute reprints for Governmental purposes notwithstanding any copyright notation thereon. The views and conclusions contained herein are those of the authors and should not be interpreted as necessarily representing the official policies or endorsements, either expressed or implied, of Air Force Research Laboratory, the Air Force Research Laboratory or the U.S. Government.

## Acknowledgments

The authors would like to thank NextFlex, specifically, Arthur C. Wall for their support throughout the project. Additionally, the authors acknowledge Steven M. Handler MD, PHD, Associate Chief of Staff for Geriatrics and Extended Care, Lisa McDade, Nurse manager, and Jamie Vaughn, Associate Chief Nurse, Community Living Center for providing feedback during the writing process of the manuscript.

## Conflict of interest

JCS, TL, SA, JP, LP, AS, and CB were employed by Contamination Source Identification, LLC. RL and JW are owners of Contamination Source Identification, LLC. AS also serves as a consultant for AIONX®.

The remaining authors declare that the research was conducted in the absence of any commercial or financial relationships that could be construed as a potential conflict of interest.

## Publisher’s note

All claims expressed in this article are solely those of the authors and do not necessarily represent those of their affiliated organizations, or those of the publisher, the editors and the reviewers. Any product that may be evaluated in this article, or claim that may be made by its manufacturer, is not guaranteed or endorsed by the publisher.
