# Supplementary material for: cleanSURFACES® intervention reduces microbial activity on surfaces in a senior care facility
Source: Front Cell Infect Microbiol. 2022 Nov 9;12:1040047. doi: 10.3389/fcimb.2022.1040047 (PMC9682068; doi:10.3389/fcimb.2022.1040047)
Supplement: Supplementary file 1 [file DataSheet_1.docx]

Supplementary Material


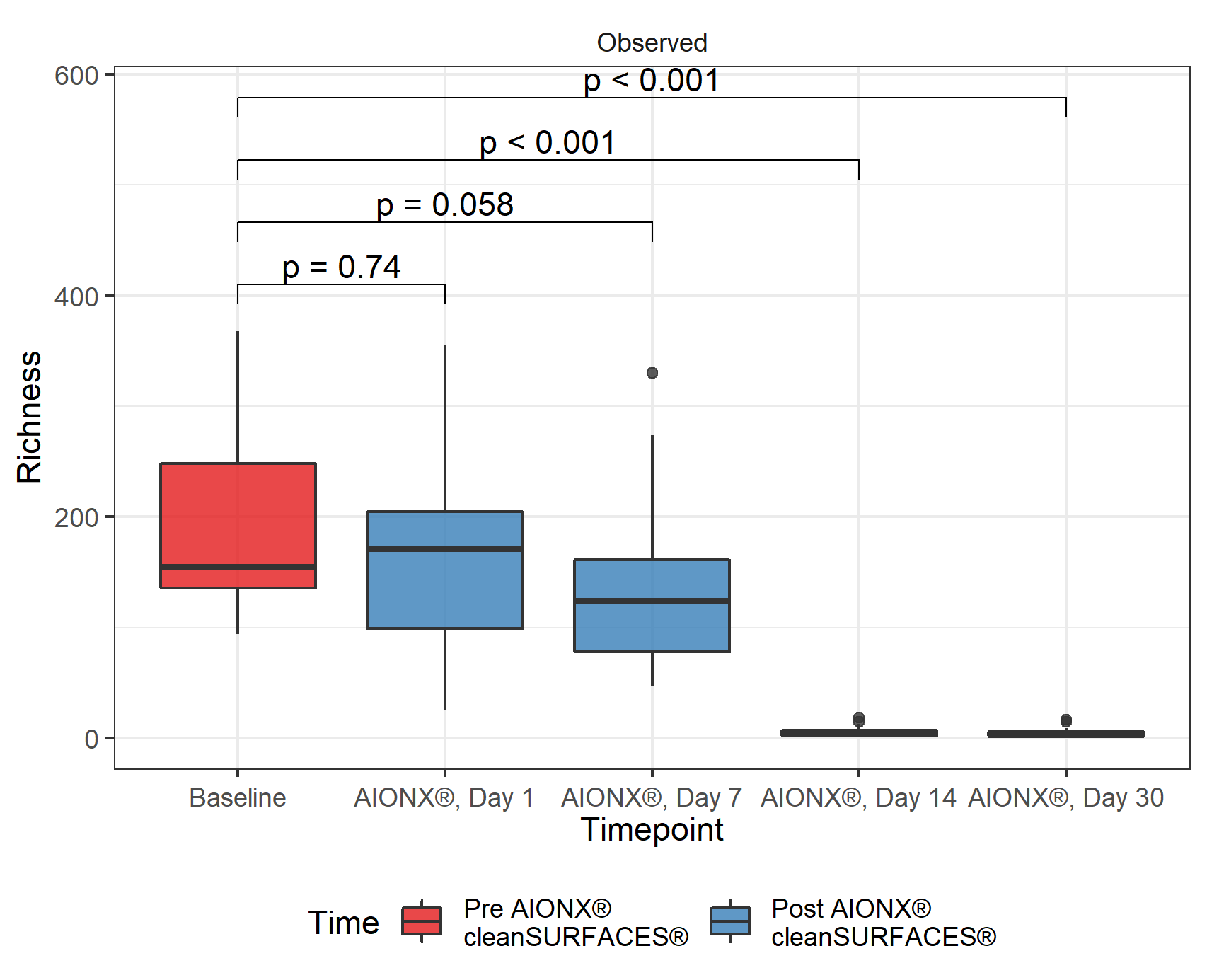


Supplemental Figure 1. Alpha Diversity during cleanSURFACES® Intervention. The number of unique taxa observed more than twice in each sample are reported. Pairwise Wilcoxon tests with a Holm correction were performed against baseline.


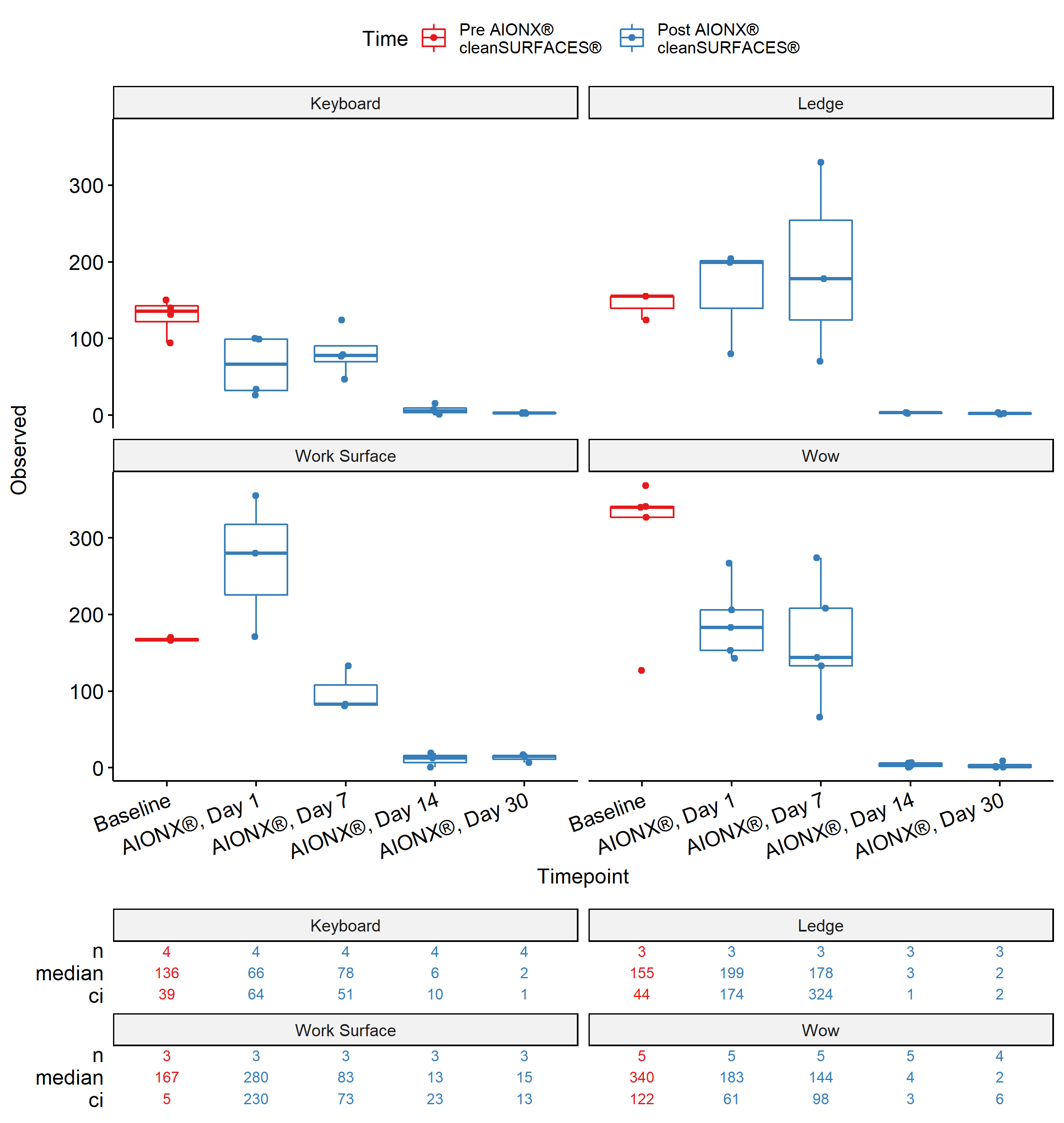


**Supplemental Figure 2.** Alpha Diversity by Surface during cleanSURFACES® Intervention. The number of unique taxa observed more than twice in each sample are reported, along with summary statistics.


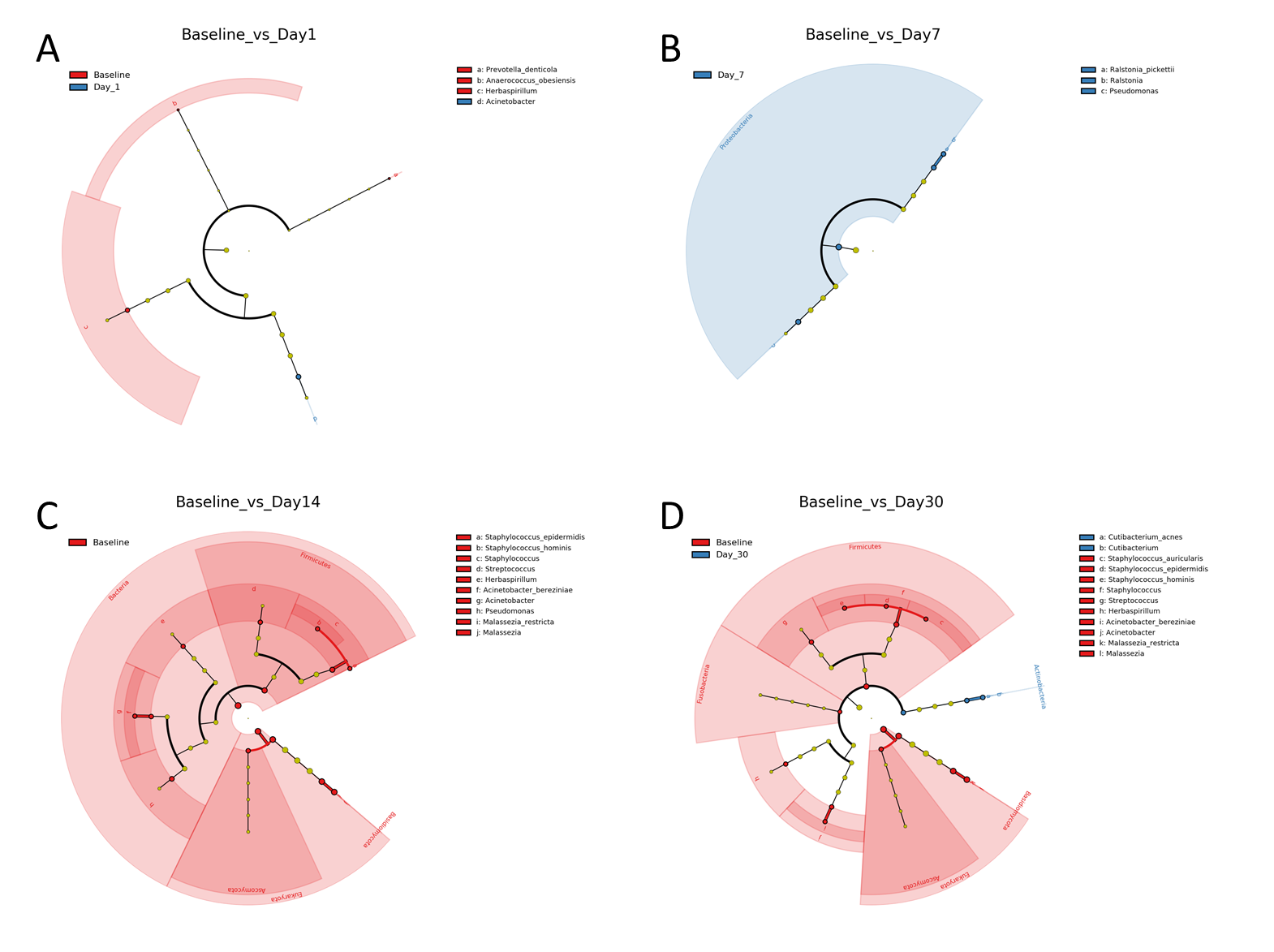


**Supplemental Figure 3**. Differential Taxa (Domain, Phylum, Genus, and Species levels) identified by LEfSe timepoint vs Pre-AIONX® cleanSURFACES® intervention analysis. The taxa shown were significantly (Kruskal-Wallis, p ≤ 0.05 and log (LDA)≥1.5) more active in Baseline samples. Taxa that were more active in Baseline are shown in red, and taxa that were more active in one of the later timepoints are shown in blue.


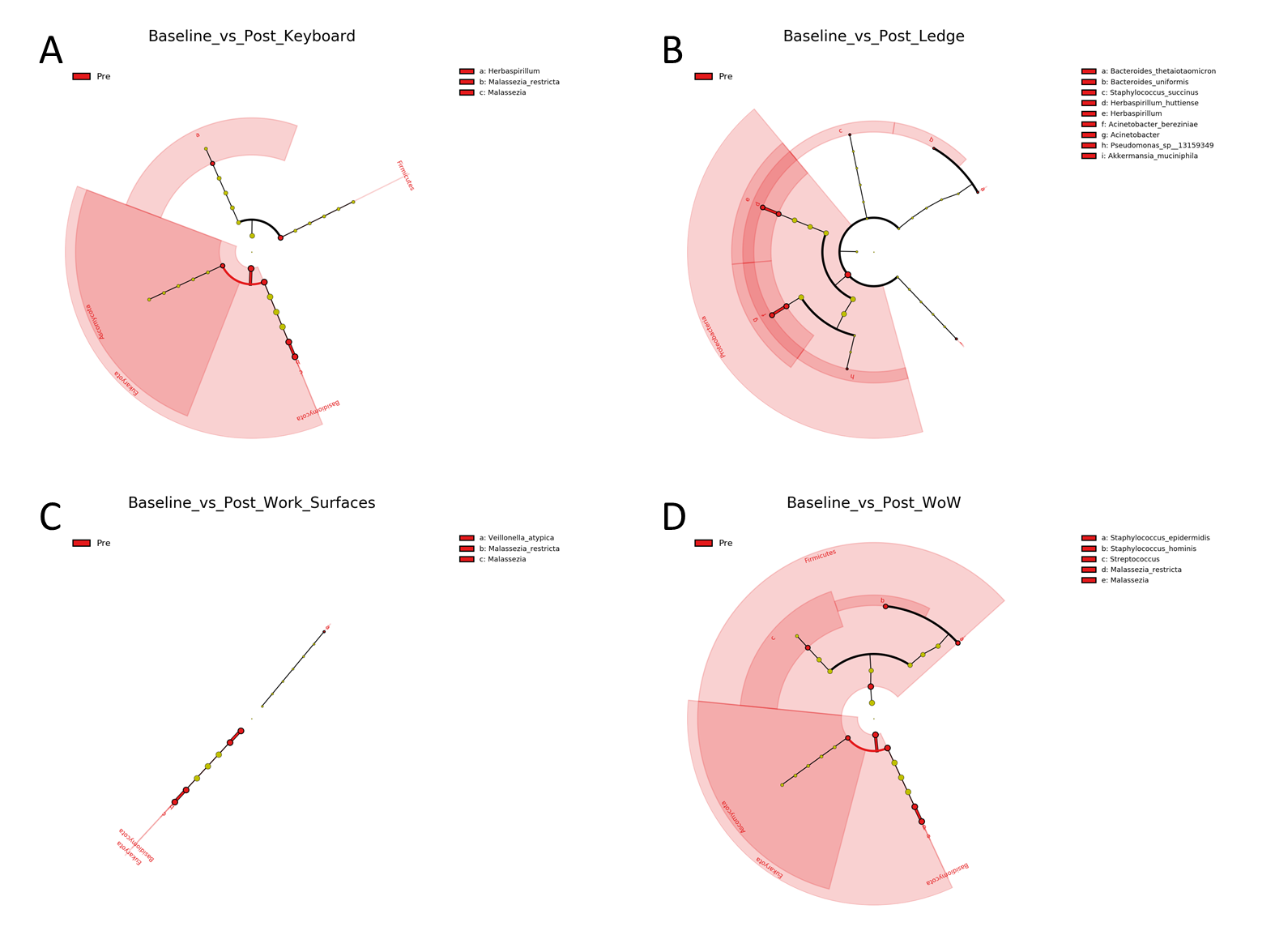
**Supplemental Figure 4**. Differential Taxa (Domain, Phylum, Genus, and Species levels) identified by Lefse Pre vs Post-AIONX® cleanSURFACES® intervention within each surface analysis. The taxa shown were significantly (Kruskal-Wallis, p ≤ 0.05 and log (LDA)≥1.5) more active in Baseline samples. Taxa that were more active in Baseline are shown in red. Like the overall Pre vs. Post- AIONX® cleanSURFACES® comparison, no differential taxa were identified in the Post group.


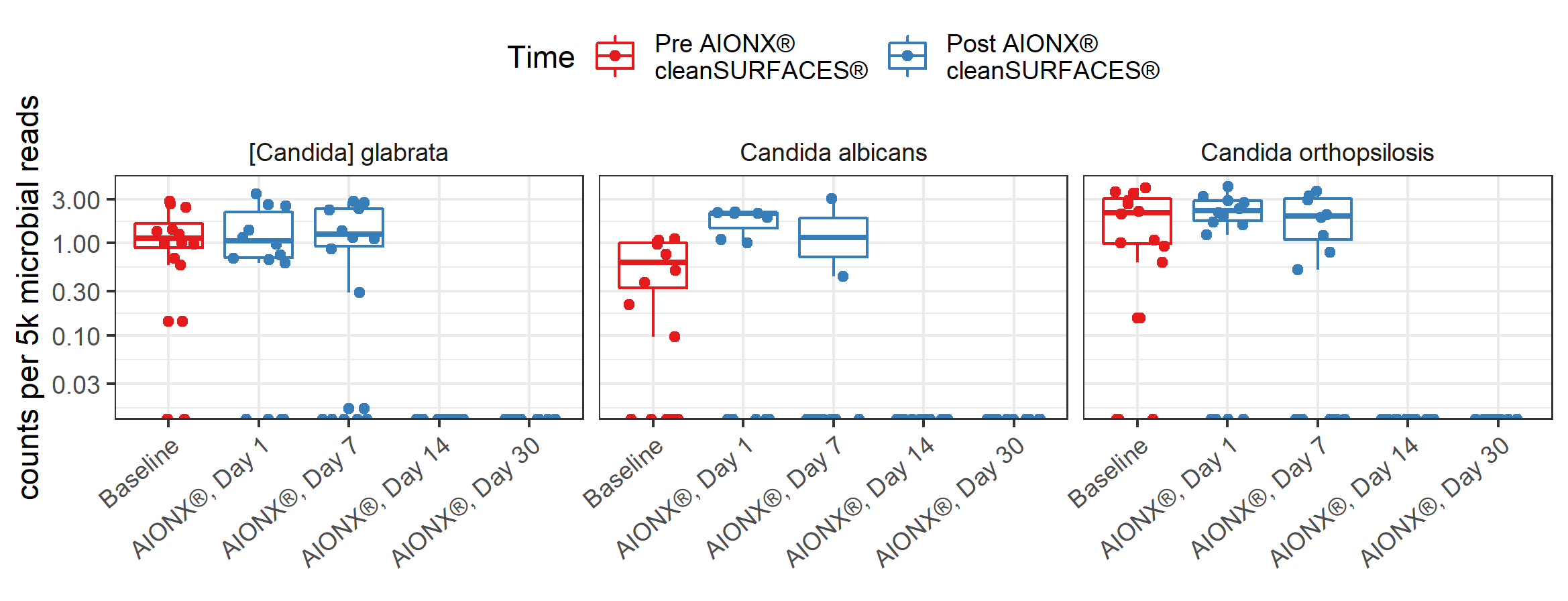
**Supplemental Figure 5.** Normalized counts of three *Candida* spp. during cleanSURFACES® intervention.

**Supplemental Table 1**. Per-sample metadata with sequencing depths and microbial read counts.

| Area/Room Location | Surface Type | Timepoint | Reads | Classified Reads |
| --- | --- | --- | --- | --- |
| Main Charting Area | Keyboard | Day -1 (Baseline) | 154751 | 15232 |
| Main Charting Area | Work Surface | Day -1 (Baseline) | 498673 | 22805 |
| Main Charting Area | Keyboard | Day -1 (Baseline) | 558322 | 82565 |
| Main Charting Area | Work Surface | Day -1 (Baseline) | 408556 | 46927 |
| Main Charting Area | Keyboard | Day -1 (Baseline) | 394076 | 63728 |
| Main Charting Area | Work Surface | Day -1 (Baseline) | 947617 | 106985 |
| Main Charting Area | Keyboard | Day -1 (Baseline) | 1294713 | 150593 |
| Hall B Liberty Place | Med Cart | Day -1 (Baseline) | 292615 | 25549 |
| Hall C Unity Way | Med Cart | Day -1 (Baseline) | 317456 | 16600 |
| Lounge | Wow | Day -1 (Baseline) | 940317 | 87410 |
| Lounge | Wow | Day -1 (Baseline) | 1947991 | 150752 |
| Lounge | Wow | Day -1 (Baseline) | 1783476 | 194372 |
| Main Charting Area | Ledge | Day -1 (Baseline) | 1005758 | 241199 |
| Main Charting Area | Ledge | Day -1 (Baseline) | 470320 | 65593 |
| Main Charting Area | Ledge | Day -1 (Baseline) | 6670943 | 2672609 |
| Hall C | Wow | Day -1 (Baseline) | 1555815 | 214328 |
| Hall D | Wow | Day -1 (Baseline) | 1496136 | 196301 |
| Main Charting Area | Keyboard | Day 1 | 756128 | 45670 |
| Main Charting Area | Work Surface | Day 1 | 1884248 | 231078 |
| Main Charting Area | Keyboard | Day 1 | 237016 | 52363 |
| Main Charting Area | Keyboard | Day 1 | 1540279 | 276200 |
| Main Charting Area | Work Surface | Day 1 | 1695984 | 95073 |
| Main Charting Area | Work Surface | Day 1 | 938250 | 52247 |
| Main Charting Area | Keyboard | Day 1 | 466939 | 35469 |
| Hall B Liberty Place | Med Cart | Day 1 | 1022605 | 103555 |
| Hall C Unity Way | Med Cart | Day 1 | 460835 | 25565 |
| Lounge | Wow | Day 1 | 522140 | 27154 |
| Lounge | Wow | Day 1 | 747913 | 37081 |
| Lounge | Wow | Day 1 | 1751933 | 105392 |
| Main Charting Area | Ledge | Day 1 | 521617 | 34460 |
| Hall D | Wow | Day 1 | 738116 | 23246 |
| Main Charting Area | Ledge | Day 1 | 850189 | 51033 |
| Hall C | Wow | Day 1 | 970244 | 57903 |
| Main Charting Area | Ledge | Day 1 | 104798 | 4822 |
| Main Charting Area | Keyboard | Day 7 | 526750 | 1285 |
| Main Charting Area | Work Surface | Day 7 | 513841 | 4019 |
| Main Charting Area | Keyboard | Day 7 | 542794 | 50817 |
| Main Charting Area | Work Surface | Day 7 | 1164781 | 19643 |
| Main Charting Area | Keyboard | Day 7 | 700409 | 5738 |
| Main Charting Area | Work Surface | Day 7 | 1218107 | 5137 |
| Main Charting Area | Keyboard | Day 7 | 1287543 | 19098 |
| Hall B Liberty Place | Med Cart | Day 7 | 666185 | 1809 |
| Hall C Unity Way | Med Cart | Day 7 | 2886984 | 77894 |
| Lounge | Wow | Day 7 | 1161394 | 68625 |
| Lounge | Wow | Day 7 | 10746261 | 1229716 |
| Lounge | Wow | Day 7 | 627346 | 36640 |
| Main Charting Area | Ledge | Day 7 | 4861124 | 338998 |
| Main Charting Area | Ledge | Day 7 | 1407508 | 124129 |
| Main Charting Area | Ledge | Day 7 | 538778 | 17892 |
| Hall D | Wow | Day 7 | 701668 | 3059 |
| Hall C | Wow | Day 7 | 820026 | 16378 |
| Main Charting Area | Keyboard | Day 14 | 31210 | 1 |
| Main Charting Area | Work Surface | Day 14 | 1370830 | 127 |
| Main Charting Area | Keyboard | Day 14 | 1130056 | 48 |
| Main Charting Area | Work Surface | Day 14 | 2703 | 0 |
| Main Charting Area | Keyboard | Day 14 | 29280 | 5 |
| Main Charting Area | Work Surface | Day 14 | 1074626 | 197 |
| Main Charting Area | Keyboard | Day 14 | 1568189 | 127 |
| Hall B Liberty Place | Med Cart | Day 14 | 1604374 | 72 |
| Hall C Unity Way | Med Cart | Day 14 | 61087 | 39 |
| Lounge | Wow | Day 14 | 42183 | 3 |
| Lounge | Wow | Day 14 | 21566 | 1 |
| Lounge | Wow | Day 14 | 1103519 | 51 |
| Main Charting Area | Ledge | Day 14 | 29891 | 3 |
| Main Charting Area | Ledge | Day 14 | 228352 | 7 |
| Main Charting Area | Ledge | Day 14 | 20634 | 1 |
| Hall C | Wow | Day 14 | 753575 | 29 |
| Hall D | Wow | Day 14 | 940966 | 28 |
| Main Charting Area | Keyboard | Day 30 | 57399 | 1 |
| Main Charting Area | Work Surface | Day 30 | 1137021 | 39 |
| Main Charting Area | Keyboard | Day 30 | 39763 | 7 |
| Main Charting Area | Work Surface | Day 30 | 628423 | 198 |
| Main Charting Area | Keyboard | Day 30 | 20717 | 1 |
| Main Charting Area | Work Surface | Day 30 | 207923 | 178 |
| Main Charting Area | Keyboard | Day 30 | 44829 | 1 |
| Hall B Liberty Place | Med Cart | Day 30 | 35168 | 1 |
| Hall C Unity Way | Med Cart | Day 30 | 65107 | 1 |
| Lounge | Wow | Day 30 | 1116758 | 75 |
| Lounge | Wow | Day 30 | 30065 | 2 |
| Main Charting Area | Ledge | Day 30 | 34743 | 2 |
| Main Charting Area | Ledge | Day 30 | 39327 | 12 |
| Main Charting Area | Ledge | Day 30 | 32903 | 0 |
| Hall C | Wow | Day 30 | 299532 | 0 |
| Hall D | Wow | Day 30 | 84084 | 1 |

**Supplemental Table 2.** Comparison of annotated microbial sequences in this study compared to our previous study of cleanSURFACES® application (Chen See and Ly et al. 2021).

| Metric | Study | N | Median | Mean | sd |
| --- | --- | --- | --- | --- | --- |
| ERCC | Pitt-VA | 91 | 3178 | 114894.802 | 310688.552 |
| ERCC | UPMC | 111 | 5309017 | 5191605.225 | 1396399.608 |
| Filtered | Pitt-VA | 91 | 666185 | 933798.802 | 1411377.958 |
| Filtered | UPMC | 111 | 5597907 | 5438223.207 | 1400578.094 |
| Microbial | Pitt-VA | 91 | 15232 | 84841.846 | 309022.555 |
| Microbial | UPMC | 111 | 9252 | 20445.658 | 35606.168 |
| Raw | Pitt-VA | 91 | 962790 | 1493389.253 | 1841504.473 |
| Raw | UPMC | 111 | 6766449 | 6492338.775 | 1529754.369 |

**Supplemental Table 3.** The contributions to mean Jaccard distance between samples due to turnover (gain of taxa) and nestedness (loss of taxa).

| Name | Value |
| --- | --- |
| Mean turnover | 0.185 |
| Mean nestedness | 0.608 |
| Mean Jaccard | 0.793 |
| Fraction attributed to turnover | 0.233 |
| Fraction attributed to nestedness | 0.767 |

**References**

Chen See, J., Ly, T., Shope, A., Bess, J., Wall, A., Komanduri, S., et al. (2021). A Metatranscriptomics Survey of Microbial Diversity on Surfaces Post-Intervention of cleanSURFACES® Technology in an Intensive Care Unit. *Front. Cell. Infect. Microbiol.* 0. doi:10.3389/fcimb.2021.705593.
